# Complete genome sequence of *Klebsiella pneumoniae* RX.G5M15, a methanol-metabolizing strain recovered from the sole of a shoe

**DOI:** 10.1128/mra.00451-24

**Published:** 2024-08-20

**Authors:** D. Y. Xu, K. M. Leung, G. K. K. Lai, F. C. C. Leung, S. D. J. Griffin

**Affiliations:** 1 Shuyuan Molecular Biology Laboratory, The Independent Schools Foundation Academy, Hong Kong SAR, China; Indiana University, Bloomington, Bloomington, Indiana, USA

**Keywords:** *Klebsiella pneumoniae*, methanol, methylotrophs

## Abstract

The methanol-metabolizing strain *Klebsiella pneumoniae* RX.G5M15 was isolated from the sole of a shoe in Hong Kong. Its complete genome, a single chromosome and two plasmids totaling 5,381,940 bp (G+C 57.43%), was established through the hybrid assembly.

## ANNOUNCEMENT

*Klebsiella pneumoniae* is a widely distributed Gram-negative species, often found as a gut commensal and as a plant growth-promoting rhizobacterium (1, 2). Contrastingly, it is also a Priority 1 ESKAPE pathogen (3), intrinsically resistant via a *blaSHV* beta-lactamase (4) and easily exchanging virulence and resistance genes (5). This genomic plasticity produces varied pathogenicity even within a single clonal group (6). Here, methanol-utilization – a function common in plant-associated microbes (7, 8) – was used as part of a screen for potentially kingdom-crossing species (9), isolating RX.G5M15 from the sole of a shoe, a known vehicle for transmission in healthcare settings (10).

A sterile disposable loop applied to the sole of a shoe (2021-11-08; 22.263738 N, 114.129972 E, Hong Kong: Pokfulam) was used to inoculate 1 mL of a minimal salts medium (11) containing 3% vol*:*vol methanol (MSM-MeOH) as the only carbon source. After incubation for 5 days with shaking, 100 µL of the mixture was spread onto Luria agar (12), and, following overnight growth, selected colonies were passaged to purity on the same medium. Isolates were subsequently reassessed for growth in MSM-MeOH by plate counts of aliquots withdrawn daily over 5 days. A single colony of RX.G5M15, the isolate showing the strongest growth in MSM-MeOH, was spread on Luria agar and incubated for 24 h before harvesting for DNA extraction (DNeasy PowerSoil Pro kit, QIAGEN GmbH). UV absorbance (BioDrop µLITE, Biochrom Ltd., UK) and PicoGreen assays (13) were used for DNA quality/quantity evaluations. All agar incubations were conducted at 27°C.

Paired-end short-read sequencing libraries were prepared using a NexteraXT DNA Library Preparation Kit and sequenced via the Illumina MiSeq platform using v3 chemistry (2 × 300 bp). A total of 1,708,149 raw reads (mean length 247 bp) (SRX24234014) were quality-filtered and trimmed using TrimGalore v0.6.7 (https://github.com/FelixKrueger/TrimGalore) (stringency: 3; -e: 0.2), producing 813,438 read pairs (mean length 215 bp, total ~349 Mbp). Long-read libraries, prepared from the same extracted DNA using the Rapid Barcoding Kit SQK-RBK004 without size selection, were sequenced via Oxford Nanopore’s Spot-ON Flow Cell (vR9.4.1), MinION sequencer, and MinKNOW v20.06.2 software, with base-calling by Guppy v6.1.5 (14). The final long-read data set of 251,151 raw reads (mean length 6,526 bp) (SRX24234015) was trimmed by Filtlong v0.2.1 (https://github.com/rrwick/Filtlong) (min_length: 2,000; target_bases: 500 Mbp; length_weight: 10), selecting 145,943 reads (mean length 10,107 bp, N50 12,420; total ~1.48 Gbp) to scaffold the assembly. Default parameters were used for all software unless otherwise specified.

Hybrid assembly, overlap removal, and sequence rotation were performed by Unicycler v0.4.8-beta (15) yielding a single chromosome and two circular plasmids (see Table 1) with mean coverage 275× [judged 98.77% complete with 0.74% contamination by CheckM v1.2.2 (16)], which were submitted to NCBI PGAP v6.4 (17) for annotation. RX.G5M15 shares an average nucleotide identity of 99.28% (18) with type-strain *Klebsiella pneumoniae* subsp. *pneumoniae* HS11286 (CP003200).

**TABLE 1:** Assembly data for RX.G5M15

Replicon	GenBank accession	Size/bp	G+C%	Topology	Rotated to begin	CDs^a^	Pseudogenes^a^
Chromosome	CP118936	5,225,085	57.60	Circular	B5XT51 (*dnaA*)	4,937	74
Plasmid pRX.G5M15_1	CP118937	152,213	51.84	Circular	Q6U5J6 (*repA*)	173	19
Plasmid pRX.G5M15_2	CP118938	4,642	43.90	Circular	Not rotated	5	0

^a^ Predicted by NCBI PGAP v6.4 (14).

KEGG mapping in BV-BRC v.3.35.5 (19) predicts methanol assimilation via a glutathione-based formaldehyde-detoxification system (20). Plasmids pRX.G5M15_1 and pRX.G5M15_2 were classified by the KpVR web-based tool as types IncFIB(K)_8[CladeI] and Col440I, respectively, with the small plasmid encoding an anti-phage dCTP deaminase (21) at locus PX704_26170. Kleborate in Pathogenwatch (22, 23) determined no virulence loci (see report for SAMN33453558).

## Data Availability

Sequencing and assembly data for *Klebsiella pneumoniae* RX.G5M15 are available through NCBI under BioProject PRJNA939005 (see also Table 1).
